# Spatial epidemiologic trends of cutaneous leishmaniasis in Rio de Janeiro State, Southeast Brazil, 2001–2020

**DOI:** 10.1590/S1678-9946202567023

**Published:** 2025-04-04

**Authors:** Tayana Patrícia Santana Oliveira de Sá, Sandro Javier Bedoya-Pacheco, Rafael Ramalho Cunha-e-Silva, Alex de Oliveira Vasconcelos, Mônica de Avelar Figueiredo Mafra Magalhães, Cristina Maria Giordano Dias, Juliana Gonçalves dos Reis, Liliane de Fátima Antonio Oliveira, Andreza Pain Marcelino, Maria Inês Fernandes Pimentel

**Affiliations:** 1 Fundação Oswaldo Cruz, Instituto Nacional de Infectologia Evandro Chagas, Laboratório de Pesquisa Clínica e Vigilância em Leishmanioses, Rio de Janeiro, Rio de Janeiro, Brazil; 2 Fundação Oswaldo Cruz, Escola Nacional de Saúde Pública Sérgio Arouca, Departamento de Epidemiologia e Métodos Quantitativos em Saúde, Rio de Janeiro, Rio de Janeiro, Brazil; 3 Fundação Oswaldo Cruz, Instituto de Comunicação e Informação Científica e Tecnológica em Saúde, Rio de Janeiro, Rio de Janeiro, Brazil; 4 Secretaria de Estado de Saúde do Rio de Janeiro, Rio de Janeiro, Rio de Janeiro, Brazil

**Keywords:** Cutaneous leishmaniasis, Mucosal leishmaniasis, Epidemiology, Cutaneous leishmaniasis composite indicator

## Abstract

Cutaneous leishmaniasis (CL) has substantial epidemiological and clinical differences depending on host characteristics, *Leishmania* species and geographic areas. CL in Rio de Janeiro State was evaluated. Mandatory notifications of confirmed cases from 2001–2020 were analyzed considering sociodemographic and clinical variables, temporal trends, the Cutaneous Leishmaniasis Composite Indicator (CLCI) for each affected city in relation to the total of affected cities in the state, and each triennium from the beginning to the end of the time series (2001–2003, 2002–2004 until 2018–2020, sequentially). The number of cases decreased over time. High average incidence rates occurred in contiguous cities from the Southernmost to the Northernmost regions of Rio de Janeiro State, following areas where the Atlantic Forest persists. The CLCI showed temporal variations in the intensity of the risk of CL in the affected cities. Rio de Janeiro city was the only one with intense or very intense risk of CL throughout the studied period. The disease predominantly affected people residing in urban areas and in the middle age groups (20–59 years). CL also predominated in males, regardless of age. The mucosal clinical form was also significantly associated with men, with an 80% chance of them being more affected than women. Regarding diagnostic tests, the Leishmanin Skin Test showed higher positivity than the direct parasitological exam and the histopathological exam. No differences regarding cure between sexes were found. This study may guide control actions in areas where they are most needed in Rio de Janeiro State.

## INTRODUCTION

Cutaneous leishmaniasis (CL) is a neglected tropical disease, and Brazil was responsible for more than 37% of cases in the Americas in recent decades^1^. The predominant species of protozoa that cause CL in Brazil are Leishmania *(Viannia) braziliensis*, dispersed throughout the country; *Leishmania (Viannia) guyanensis*, mainly to the North of the Amazon Basin; and *Leishmania (Leishmania) amazonensis*, which is also dispersed throughout Brazil, although predominantly in the Amazon biome. Protozoa are transmitted by infected females of some sandfly species during blood feeding. The disease is essentially considered a zoonosis, in which wild and synanthropic animals (such as rodents and marsupials) are the main reservoirs, while humans and domesticated animals are considered accidental hosts^2^.

CL is present in all states and five regions of Brazil^3^. In Rio de Janeiro, similar to other states in the Southeast region (Minas Gerais, Espirito Santo and Sao Paulo), the disease has low endemicity, and the predominant parasitic species is *Leishmania (Viannia) braziliensis* ^4,5^. The sandfly species most implicated in transmission in Rio de Janeiro State is Nyssomyia intermedia, due to its anthropophilic feeding habit and adaptation around human dwellings. However, other species, such as Migonemyia migonei, which feed mainly in dogs, may also be occasionally involved in human transmission^6^.

In addition to CL, the disease can also manifest as mucosal leishmaniasis (ML), which is related almost exclusively to the infection with *Leishmania (Viannia) braziliensis in Brazil* ^2^. Two other unusual forms also occur: disseminated leishmaniasis and diffuse leishmaniasis^2^. ML is recorded in 2.8% to 3.5% of the Northeast, 3% to 7% of the North, 8.6% of the Central-West, and 12% to 23% of the South and Southeast^7^. The highest rates of ML were observed in regions with the lowest incidence of CL, namely, the South and Southeast^7,8^.

The epidemiological surveillance of CL is traditionally carried out by checking the number of cases and the incidence rate per 100,000 inhabitants in a territory; other variables such as sex and age group are also analyzed. However, the use of isolated indexes to assess CL endemicity has limitations. Brazil, for example, has a high number of CL cases compared to other countries in America, but low incidence rate due to its large population^9^. In recent years, the Pan American Health Organization (PAHO) and the Brazilian Ministry of Health have evaluated the risk of CL using the Cutaneous Leishmaniasis Composite Indicator (CLCI), which can be used at various levels^10-12^.

To analyze the CL scenario in Rio de Janeiro State in the first two decades of the 21^st^ century, data from mandatory notifications in the System of Information of Diseases of Notification (SINAN), as well as from population and territorial information from affected cities, were used to develop a clinical, epidemiological, temporal, and spatial study.

## MATERIALS AND METHODS

### Study design

A retrospective study was carried out with spatial and temporal analyses of the incidence of CL, complemented with analyses of demographic and clinical characteristics. This study was approved by the Research Ethics Committee of the Instituto Nacional de Infectologia Evandro Chagas, Fundacao Oswaldo Cruz, Rio de Janeiro, Brazil, CAAE 15986719.2.0000.5262, by technical report Nº 3.507.409 released on August 14, 2019.

### Study area

Rio de Janeiro State, encompassing 92 cities, with a population of 15,989,929 people (2010 census). It is the third Brazilian state with the largest number of inhabitants^13^, and its geographic area is 43,750,425 square kilometers^2 14^.

### Study population

The studied population were individuals with CL residing in Rio de Janeiro State, notified in the SINAN database between 2001 and 2020 and registered in the files provided by the Health Department of Rio de Janeiro State. Collected information included clinical forms (cutaneous or mucosal), diagnostic laboratory tests, treatment, outcome, sex, age, and address (city, neighborhood, street, house number). Population data to construct incidence rates were obtained from the Brazilian Institute of Geography and Statistics (IBGE), 2010 census, and estimates for the years in which censuses were not performed (2001–2009 and 2011–2020)^13^.

### Analysis procedures

Temporal analysis of cases: the time interval for time series analysis was annual.

Incidence rates: incidence rates in different cities were calculated per 100,000 inhabitants.

Composite indicator for stratification of cutaneous leishmaniasis transmission risk (CLCI): CLCIs per triennium were overlapping and sequential (2001-2003, 2002-2004, 2003–2005, 2004–2006, 2005–2007, 2006-2008, 2007-2009, 2008–2010, 2009–2011, 2010-2012, 2011-2013, 2012–2014, 2013–2015, 2014–2016, 2015–2017, 2016-2018, 2017–2019, and 2018–2020). They were built to increase consistency and adjust for sudden variations over the years. They were also calculated for each affected city (i.e., cities with notified residents with CL infection), in each triennium, using the Excel program. The objective was knowing areas at risk of CL occurrence, integrating the information contained in the numbers of cases and the incidence rates. The methodology used to calculate the CLCI was according to the following steps^2,15^:

- Number of CL cases by city of residence (triennium): the number of cases (N) for the three years of each triennium (N_3-year_) for each affected city were added and divided by three. For example, for city 1 in the 2001–2003 triennium:
$$\text{N}1_{3\text{-year}}=\frac{\text{N}1_{2001}+\text{N}1_{2002}+\text{N}1_{2003}}{3}$$
- Incidence rates (triennium): the incidence rates for each triennium (I_3-year_) were calculated for each affected city per 100,000 inhabitants, added together and divided by three. For example, for city 1 in the period of 2001–2003:
$${\text{Il}}_{3\text{-year}}=\frac{\text{I}1_{2001}+{\text{Il}}_{2002}+{\text{Il}}_{2003}}{3}$$
- General average of the number of cases, of incidence rates, of standard deviations of the number of cases, and of standard deviation of the incidence rates for all affected cities in each triennium: the general average of the number of cases of all affected cities (N1, N2, N3, …, NX) was calculated (for example, X affected cities in the triennium, GAN_3-year_ below), the general average of the incidence rates of all affected cities (I1, I2, I3, …, IX) (for example, X affected cities in the triennium, GAI_3-year_ below), the general average of standard deviations of the number of cases of all affected cities (for example, X affected cities in the triennium, GASDN_3-year_ below), and the general average of standard deviations of incidence rates for all affected cities (for example, X affected cities in the triennium, GASDI_3-year_ below) in each triennium.
$${\text{GAN}}_{3\text{-year}}=\frac{\text{N}1_{3\text{-year}}+\text{N}2_{3\text{-year}}+\text{N}3_{3\text{-year}}+\ldots +{\text{NX}}_{3\text{-year}}}{X}$$
$${\text{GAI}}_{3\text{-year}}=\frac{\text{I}1_{3\text{-year}}+\text{I}2_{3\text{-year}}+\text{I}3_{3\text{-year}}+\ldots +{\text{IX}}_{3\text{-year}}}{\text{X}}$$
$${\text{GASDN}}_{3\text{-year}}=\frac{{\text{SDN1}}_{3\text{-year}}+{\text{SDN}}_{3\text{-year}}+{\text{SDN}}_{3\text{-year}}+\ldots +{\text{SDNX}}_{3\text{-year}}}{\text{X}}$$
$${\text{GASDI}}_{3\text{-year}}=\frac{{\text{SDI1}}_{3\text{-year}}+{\text{SDI}}_{3\text{-year}}+{\text{SDI}}_{3\text{-year}}+\ldots +{\text{SDIX}}_{3\text{-year}}}{\text{X}}$$
- Normalized index of number of cases: for each triennium, the normalized index of number of cases (NIN_3-year_) for an affected city was calculated by subtracting the number of cases of each triennium for the affected city from the general average number of cases for all affected cities, and dividing by the general average of the standard deviations of the number of cases in all affected cities. For example, the normalized index of the number of cases for city 1 is:
$${\text{NIN}}_{3\text{-year}}=\frac{\text{N}1_{3\text{-year}}-{\text{GAN}}_{3\text{-year}}}{{\text{GASDN}}_{3\text{-year}}}$$
- Normalized index of the incidence rate: for each triennium, the normalized index of the incidence rate (NII_3-year_) for an affected city was calculated by subtracting the incidence rate of each affected city from the general average incidence rate of all affected cities and dividing by the general average of standard deviations of incidence rates for all affected cities. For example, the normalized index of the incidence rate for city 1 is:
$${\text{NII1}}_{3\text{-year}}=\frac{{\text{I1}}_{3-\text{year}}-{\text{GAI}}_{3\text{-year}}}{{\text{GASDI}}_{3\text{-year}}}$$
- Cutaneous Leishmaniasis Composite Indicator (CLCI): finally, the CLCI was calculated for each affected city by summing the normalized index of the number of cases with the normalized index of the incidence rate. For example, for city 1, the CLCI for a triennium is:

$${\text{CLCI}}_{3\text{-year}}={\text{NIN1}}_{3\text{-year}}+{\text{NII1}}_{3\text{-year}}$$

For each city with residents reported with CL in Rio de Janeiro State, the CLCI for the triennium was categorized based on "natural cuts" that enable generating five classification strata of the transmission risk: low, medium, high, intense, and very intense.

Analysis of demographic and clinical characteristics: Age and sex, clinical forms (cutaneous and mucosal), diagnostic laboratory tests, drugs used in treatment, and outcomes were analyzed. The statistical software R (version 4.2.1, University of Auckland, Auckland, New Zealand) was used. The significance level was set at p < 0.05.

## RESULTS

### Number of cases

In Rio de Janeiro State, 2,286 cases of CL were reported during 2001–2020. The number of cases decreased over time, especially since 2006.

### Incidence

**Figure 1:**
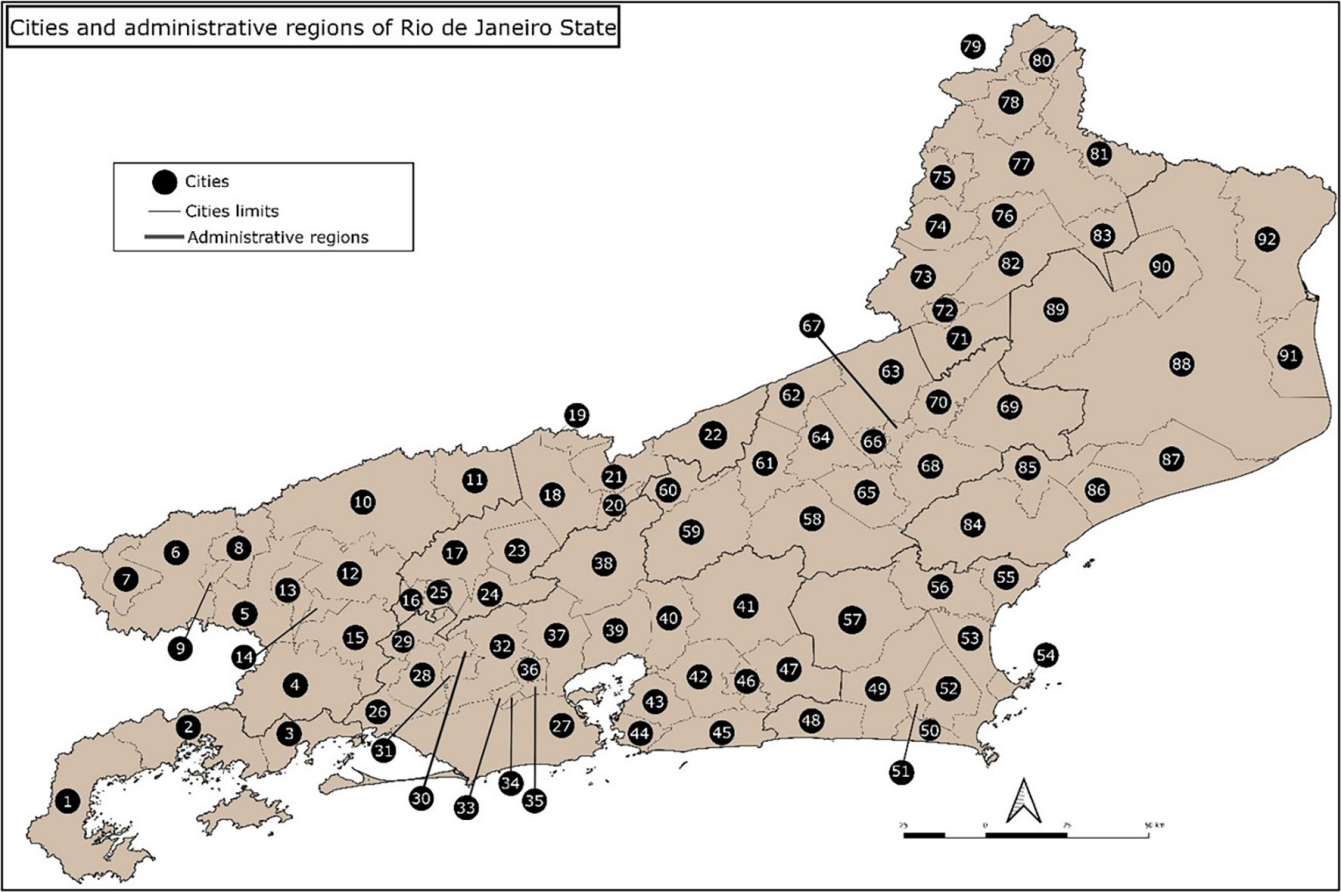
Cities of Rio de Janeiro State numbered from the left to the right, according to Table 1.

The annual incidence was 0.72 cases/100,000 inhabitants in Rio de Janeiro State from 2001–2020. In Figure 1, the 92 cities in the state are represented. Table 1 shows the cities grouped into administrative regions, their populations, geographic area, and respective incidences of CL for the studied period.

**Table 1:** Cities and administrative areas of Rio de Janeiro State regarding their populations, area in square kilometers (km^2^), and average incidence rate of cutaneous leishmaniasis in the period of 2001–2020, according to data from the Information System for Notifiable Diseases (SINAN).

Administrative region / City	Population	Area (km^2^)	Incidence
**GREEN COAST REGION**			
1. Paraty	37,533	923.24	37.57
2. Angra dos Reis	169,511	816.98	6.11
3. Mangaratiba	36,456	367.60	2.47
**PARAIBA VALLEY REGION**			
4. Rio Claro	17,425	847.10	1.72
5. Barra Mansa	177,813	548.05	0.76
6. Resende	119,769	1099.74	0.21
7. Itatiaia	28,783	240.95	0.69
8. Quatis	12,793	284.83	0.78
9. Porto Real	16,592	50.89	0.00
10. Valenca	71,843	1300.84	0.90
11. Rio das Flores	8,561	478.83	1.17
12. Barra do Pirai	94,778	584.24	0.42
13. Volta Redonda	257,803	182.13	0.23
14. Pinheiral	22,719	82.26	0.22
15. Pirai	26,314	490.22	1.52
**SOUTH-CENTER REGION**			
16. Mendes	17,935	95.31	4.46
17. Vassouras	34,410	536.31	4.07
18. Paraiba do Sul	41,084	288.18	2.68
19. Comendador Levy Gasparian	8,180	108.80	4.89
20. Areal	11,423	110.66	2.63
21. Tres Rios	77,432	323.25	1.29
22. Sapucaia	17,525	541.01	5.14
23. Paty do Alferes	26,359	314.07	0.57
24. Miguel Pereira	24,642	288.18	2.43
25. Engenheiro Paulo de Frontin	13,237	139.38	1.89
**METROPOLITAN REGION**			
26. Itaguai	109,091	282.32	1.47
27. Rio de Janeiro	6,320,446	1200.20	0.46
28. Seropedica	78,186	265.21	2.81
29. Paracambi	47,124	190.95	2.86
30. Japeri	95,492	81.68	0.89
31. Queimados	137,962	75.92	0.43
32. Nova Iguacu	796,257	520.60	0.48
33. Mesquita	168,376	41.18	0.56
34. Nilopolis	157,425	19.36	0.06
35. Sao Joao de Meriti	458,673	35.13	0.10
36. Belford Roxo	469,332	78.96	0.09
37. Duque de Caxias	855,048	467.13	0.14
38. Petropolis	295,917	791.93	0.19
39. Mage	227,322	390.75	1.28
40. Guapimirim	51,483	358.38	1.17
41. Cachoeiras de Macacu	54,273	954.61	2.03
42. Itaborai	218,008	429.56	0.11
43. Sao Goncalo	999,728	248.40	0.04
44. Niteroi	487,562	133.75	0.11
45. Marica	127,461	361.53	1.06
46. Tangua	30,732	143.01	0.49
47. Rio Bonito	55,551	459.49	1.44
48. Saquarema	74,234	352.25	4.38
49. Araruama	112,008	638.50	0.49
50. Arraial do Cabo	27,715	151.79	0.18
51. Iguaba Grande	22,851	51.05	0.00
52. Sao Pedro da Aldeia	87,875	332.44	0.00
53. Cabo Frio	186,227	398.59	0.11
54. Armacao dos Buzios	27,560	86.06	0.18
55. Rio das Ostras	105,676	228.07	0.09
56. Casimiro de Abreu	35,347	462.87	0.99
57. Silva Jardim	21,349	937.55	0.47
**MOUNTAINOUS REGION**			
58. Nova Friburgo	182,082	935.38	0.85
59. Teresopolis	163,746	773.35	0.95
60. Sao Jose do Vale do Rio Preto	20,251	219.65	10.37
61. Sumidouro	14,900	413.39	1.34
62. Carmo	17,434	306.13	3.15
63. Cantagalo	19,830	747.32	3.28
64. Duas Barras	10,930	379.59	0.91
65. Bom Jardim	25,333	382.45	5.92
66. Cordeiro	20,430	113.05	1.71
67. Macuco	5,269	78.36	2.85
68. Trajano de Moraes	10,289	591.15	23.33
69. Santa Maria Madalena	10,321	810.96	9.69
70. Sao Sebastiao do Alto	8,895	397.21	13.49
**NORTHWEST REGION**			
71. Itaocara	22,899	433,.9	3.28
72. Aperibe	10,213	94.54	0.00
73. Santo Antonio de Padua	40,589	603.30	0.86
74. Miracema	26,843	303.40	0.56
75. Laje do Muriae	7,487	253.54	5.34
76. Sao Jose de Uba	7,003	249.84	0.71
77. Itaperuna	95,841	1106.76	1.15
78. Natividade	15,082	386.91	2.32
79. Porciuncula	17,760	291.45	1.69
80. Varre-Sai	9,475	201.93	1.06
81. Bom Jesus do Itabapoana	35,411	596.63	0.71
82. Cambuci	14,827	558.12	1.01
83. Italva	14,063	290.60	1.07
**NORTH REGION**			
84. Macae	206,728	1216.78	0.27
85. Conceicao de Macabu	21,211	338.27	0.24
86. Carapebus	13,359	304.93	0.37
87. Quissama	20,242	719.70	0.25
88. Campos dos Goytacazes	463,731	4032.74	0.11
89. Sao Fidelis	37,543	1034.82	8.39
90. Cardoso Moreira	12,600	523.05	0.79
91. Sao Joao da Barra	32,747	451.10	0.00
92. Sao Francisco do Itabapoana	41,354	1118.18	0.12

Incidence = average incidence rates of cutaneous leishmaniasis per 100,000 inhabitants, 2001^–^2020; km^2^ = square kilometers; bold capital letters = administrative regions. Sources: population from the cities of Rio de Janeiro State, Brazilian Institute of Geography and Statistics (IBGE), 2010 census^13^; areas: State Center of Statistics, Research and Government Employees Formation of Rio de Janeiro Foundation (CEPERJ)^16^. Numbers according to the map in Figure 1.

**Figure 2:**
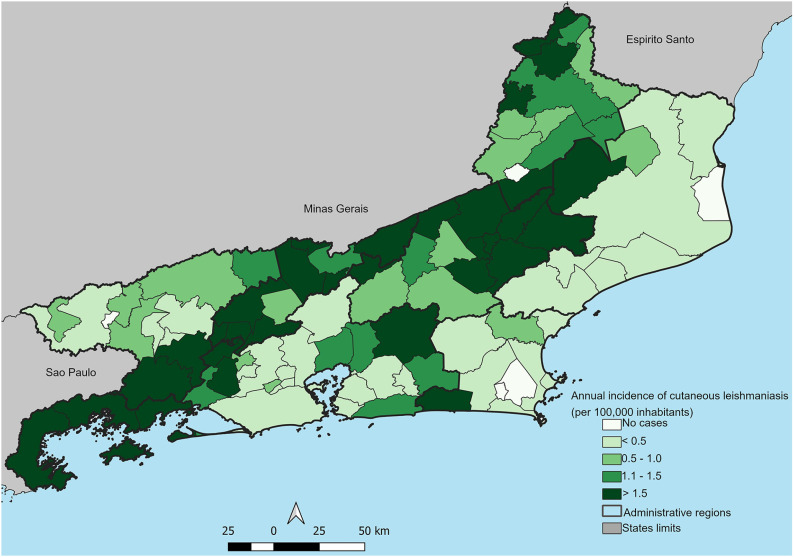
Average of the annual incidence rates per 100,000 inhabitants of cutaneous leishmaniasis in the cities of Rio de Janeiro State, 2001–2020.

CL is not homogeneously distributed across the Rio de Janeiro territory. Figure 2 shows the average annual CL incidence rates per city for the period of 2001–2020. Most cities with high incidence rates (> 1.5 cases/100,000 inhabitants) are included in an area that forms a diagonal band from Paraty city, on the Costa Verde region (number 1 in Figure 1 and Table 1), to Sao Fidelis city, North of Rio de Janeiro State (number 89 in Figure 1 and Table 1). Only five cities did not present CL cases in the studied period. Most areas with low incidence rates (< 0.5 cases/100,000 inhabitants) were found in the coastal part of the North (numbers 84–88 and 92 in Figure 1 and Table 1) and in the Coastal Lowlands (numbers 49, 50, 53–55 and 57 in Figure 1 and Table 1).

### Cutaneous Leishmaniasis Composite Indicator (CLCI)

**Figure 3:**
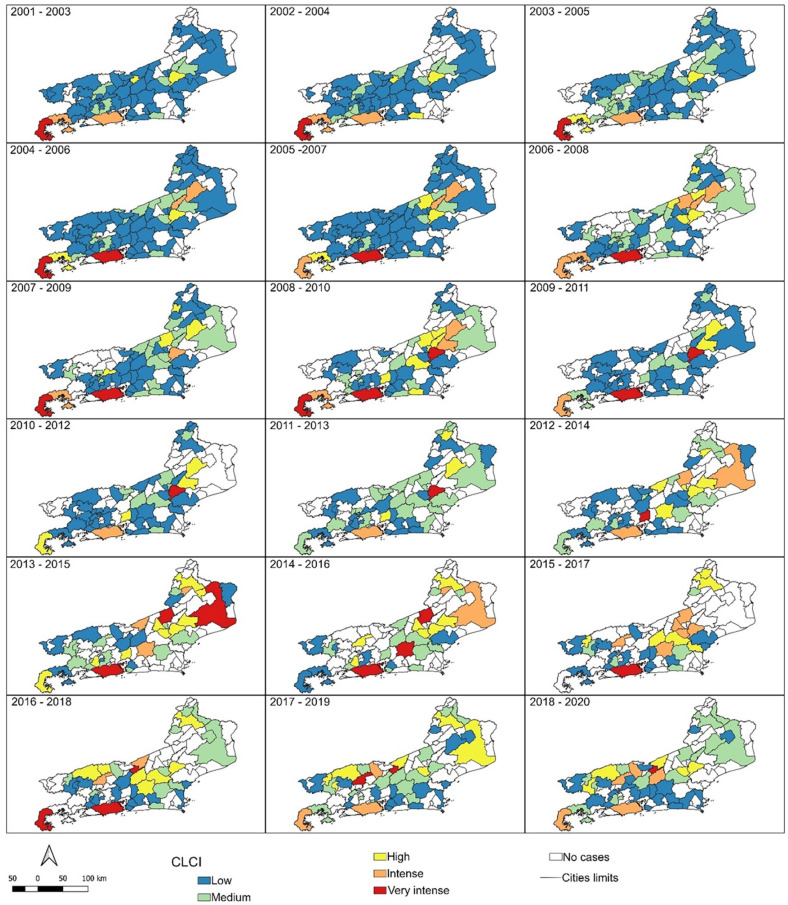
Cutaneous Leishmaniasis Composite Indicator (CLCI) for triennia (indicated at the left of the top of each map) from 2001 to 2020 in the cities of Rio de Janeiro State. Colors indicate different risk levels in the affected cities for each triennium. White means unaffected cities in the respective triennia.

CLCIs (Figure 3) of the overlapping and sequential triennia show that CL risk is concentrated in the first periods in the cities of Paraty and Angra dos Reis (numbers 1 and 2 in Figure 1 and Table 1) in the Green Coast region, and in Rio de Janeiro city (number 27 in Figure 1 and Table 1) in the metropolitan region. From 2013 onwards, the risks are concentrated in other areas (Trajano de Moraes city, number 68 in Figure 1 and Table 1), and once again concentrated in the cities of Paraty, Angra dos Reis and Rio de Janeiro. The progressive increase in cities without reported CL cases is remarkable. In the first 11 triennia (from 2001–2003 to 2011–2013) half or more of the 92 cities in Rio de Janeiro State (≥ 46 cities) were affected. From 2012–2014 onwards, the number of affected cities was always below 50% of the total, except for the last triennium in the series (2018–2020), in which 46 (50%) of the cities were affected.

### Analysis of demographic and clinical characteristics

The median age was 36.5 years (interquartile range IQR 20–51). Regarding the area of residence, most CL cases in Rio de Janeiro State between 2001 and 2020 (60.1%) were in urban areas, 33.0% were in rural areas, and 6.9% were in peri-urban areas. There was no difference between sexes in terms of the area of residence. In 212 cases, this information was not registered in the notification form.

**Figure 4:**
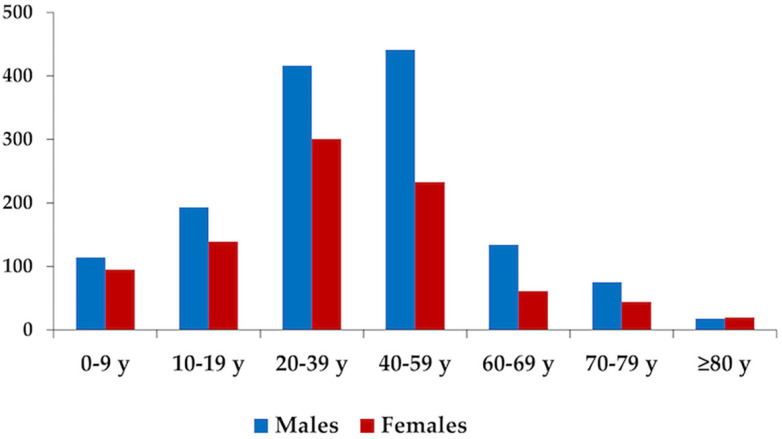
Gender according to age range of the confirmed cases of cutaneous leishmaniasis in the Information System for Notifiable Diseases (SINAN), 2001–2020. Cases of ATL in Rio de Janeiro State, data provided by the Health Department of Rio de Janeiro State; y = years.

In Figure 4, the distribution according to sex and age group for CL cases are observed. The predominant age groups were 20–39 years and 40–59 years old, and the lowest proportion of cases was found in the youngest (children under 10) and oldest (patients over 60) age groups. The largest number of cases was reported in men (60.9%), compared to women (39.1%). Men predominated over women in almost all age groups, except in older people (≥ 80 years old).

Table 2 shows the results of clinical and laboratory variables, analyzed according to sex. The cutaneous form predominates in both sexes; however, men are 80% more likely than women to present the mucosal form (p = 0.001; OR = 1.8; 95% CI = 1.333–2.464). Regarding diagnosis, none of the evaluated methods (direct parasitological examination, histopathology, and Leishmanin Skin Test [LST]) proved to be different between both sexes. LST showed a higher percentage of positive results among people who have the disease (93.2%) than direct parasitological examination (77.1%) and histopathology (41.2%). Regarding treatment, meglumine antimoniate was the first choice for more than 90% of the cases. Patients aged 60 years or more were treated in a greater proportion with amphotericin B or other medications (12.3%) compared to patients under 60 years of age (5.5%). Regarding cure, no significant difference was observed between men and women. In terms of outcome, the proportion of unreported cases was 285 (31.9%) in women and 366 (26.3%) in men. In summary, there were no significant differences between women and men regarding CL in Rio de Janeiro State, except in the clinical presentation of mucosal form, which was significantly more frequent in the latter.

**Table 2:** Prevalence rates, odds ratio, relative risk, p-value and 95% confidence intervals of clinical forms, laboratory results of the diagnostic exams, drugs used in the treatment, and clinical outcomes in females and males with cutaneous leishmaniasis in Rio de Janeiro, State, Brazil, 2001–2020.

Variables		Total		Males		Females		OR/RR	p-value	95% CI
		N	%	N	%	N	%			
**Clinical form**										
	Mucosal	226	10.1	165	12.1	61	7.1	1.8/1.2	0.001	1.333—2.464
	Cutaneous	2,001	89.9	1,198	87.9	803	92.9			
	**Total**	**2,227**	**100.0**	**1,363**	**100.0**	**864**	**100.0**			
**Direct exam**										
	Positive	668	77.1	428	77.3	240	76.9	1.0/1.0	0.907	0.732—1.417
	Negative	198	22.9	126	22.7	72	23.1			
	**Total**	**866**	**100.0**	**554**	**100.0**	**312**	**100.0**			
**Histopathology**										
	Positive	407	41.2	268	41.3	139	40.9	1.0/1.0	0.945	0.778—1.328
	Negative	582	58.2	381	58.7	201	59.1			
	**Total**	**989**	**100.0**	**649**	**100.0**	**340**	**100.0**			
**LST**										
	Positive	1,469	93.2	899	93.8	570	92.1	1.3/1.1	0.180	0.884—1.940
	Negative	108	6.8	59	6.2	49	7.9			
	**Total**	**1,577**	**100.0**	**958**	**100.0**	**619**	**100.0**			
**Drugs**										
	MA	1,740	93.4	1,097	93.9	643	92.7	1.0/0.9	0.286	0.562—1.184
	Others	122	6.6	71	6.1	51	7.3			
	**Total**	**1,862**	**100.0**	**1,168**	**100.0**	**694**	**100.0**			
**Outcome**										
	Not cured	814	35.6	468	33.6	346	38.7	0.8/0.9	0.083	0.716—1.020
	Cured	1,472	64.4	924	66.4	548	61.3			
	**Total**	**2,286**	**100.0**	**1,392**	**100.0**	**894**	**100.0**			

LST = Leishmanin skin test; MA = meglumine antimoniate. Histopathology and direct exam were considered positive when amastigotes were seen under optical microscopy; OR = odds ratio; RR = relative risk; 95% CI = 95% confidence interval.

## DISCUSSION

The number of CL cases in Rio de Janeiro State showed a decrease over the first two decades of the twenty-first century. The drop in the number of cases and incidence of CL in Brazil had already been pointed out in previous studies^17^.

The average incidence rate per city represented in Figure 2 shows a contiguity of cities with high incidence rates ranging from the Green Coast region to the North region, diagonally following the Rio de Janeiro State territory. This high-incidence area follows the state's territory where there is a greater concentration of residual Atlantic Forest, regardless of altitude (from cities at sea level such as Paraty, to cities in the mountainous region with higher altitudes such as Trajano de Moraes, with 655 meters). Centuries ago, the Rio de Janeiro State territory was greatly covered by the Atlantic Forest, which extended along most of the Eastern Brazilian coast, and which was gradually deforested as cities and populations expanded. Our results clearly show the disease reflects the spatial land mosaics existing in the Rio de Janeiro State territory, where urban areas, agricultural areas, and forest fragments interact, although the notifications refer to residences in urban areas in approximately 60% of cases.

The CLCI, used in recent years by PAHO and the Brazilian Ministry of Health, contextualizes the risk of the disease for the population considering a specific area and a unit of analysis that can be varied. For example, all of America, in terms of the first (states/provinces) or second (cities) subnational levels^1^, or Brazil, regarding the affected cities^10-12^. CLCI enables a contextualized analysis of the characteristics and peculiarities of an area, which cannot be extrapolated to other areas, unless they are included in the model. The CLCI used by the Brazilian Ministry of Health classifies affected Brazilian cities in terms of the risk of CL since the 2018–2020 period^10^. This analysis was also made available sequentially for the 2019–2021^11^ and 2020–2022 triennia^12^. In all these, each of the affected cities in Rio de Janeiro State was classified in the "low" stratum, compared to the rest of the Brazilian affected cities^10-12^. The CLCI has been used to analyze this disease by aggregating the analysis of the normalized average of cases together with the normalized average of the incidence rate into a single indicator, enabling a comparison of areas (such as cities) with different population sizes with more sensitivity, since the incidence rate calculation includes the size of the population. The objective is to identify areas that require greater attention in surveillance and control actions^1,10-12^. It has been used in some Brazilian states, such as Bahia^18^ and Ceara^19^, and has been the foundation for developing surveillance and control strategies in specific areas^20^ by identifying places at greater risk, which require more effective actions.

In this study, CLCI was used considering the affected cities within all 92 municipalities in Rio de Janeiro State. There was a substantial variation in the risk of the disease in several cities over the years. The Green Coast, metropolitan and mountainous regions stood out as having cities with intense or very intense risk in most of the triennia analyzed. Regarding cities, it was observed that Paraty, Rio de Janeiro and Trajano de Moraes (in the Green Coast, metropolitan and mountainous regions, respectively) remained at intense or very intense risk in most analyses. The same applies for other cities for a considerable part of the time, such as Sao Fidelis (in the North region) during the triennia in the middle of the analyzed 20-year period, but, subsequently, the risk decreased in this city. The CLCI clearly shows the variation in the risk of presenting CL in Rio de Janeiro State, which can increase, decrease, or even disappear momentarily, only to appear again or increase after some time. This can be explained because, despite the high urbanization, transmission conditions are maintained by the persistence of reservoirs and vectors in constant contact with the population in cities that are continually expanding by occupying previously forested areas.

The results indicate that around 60% of patients in Rio de Janeiro State lived in urban areas during the studied period. Many defend the idea that areas with lower CL endemicity in Brazil have lower rates of rural cases compared to highly endemic areas, such as the states of Acre^21^, Amazonas, and Pernambuco^22^ in the North and Northeast regions of Brazil. Some large and medium-sized cities in areas outside the North region present a substantial number of cases in the urban-rural interface around urban centers, or even in the urban area^23^, as our data for Rio de Janeiro State between 2001 and 2020 also show. Furthermore, the presence of forest fragments within some cities could be implicated in the illness of individuals residing in urban areas, with no recent history of exposure in known endemic CL areas^24^.

Approximately 61% of patients were male. In Brazil, this predominance is widely known^2^. The different CL infection rates between sexes has been the subject of constant speculation. The predominance in men leads us to consider this is due to greater exposure for work reasons, which can happen with farmers, military personnel, gold-diggers, and miners in general^22,25^. Thus, under similar exposure conditions, the number of cases between men and women would tend to be similar^26^. Some authors consider biological differences as responsible for the predominance in men^27^.

CL in Rio de Janeiro State predominated in the middle age groups, both in men and women. These results are consistent with the age pyramid according to sex for CL in Brazil^28^. Several Brazilian studies reported this predominance in adults^25,29^, which also raises the question about the importance of occupational exposure. Children and older people are naturally more restricted to their homes than adults, and adult men tend to work outside, occasionally travelling to endemic areas^25^. However, the involvement of young children under five years of age suggests that transmission occurs mainly within or near the home. This has already been pointed out by several authors^21,28^. The occurrence in older children or adolescents could be related to circulation in forests of the Amazon Basin^30^.

ML occurred in 10% of the cases and was significantly associated with males, who had approximately 80% more chance of developing it than females. The predominant occurrence of ML in men has previously been found in South America^31^. The clinical form of ML in Brazil is usually related to *Leishmania (Viannia) braziliensis* ^31^, or, more rarely, to other species. In Rio de Janeiro State, *Leishmania (Viannia) braziliensis* is almost the exclusive species causing CL^4,5^.

The diagnosis is made after analyzing epidemiological history and clinical examination, with the aid of laboratory tests. The tests for the CL diagnosis showed differences in performance: LST was positive in the highest percentages of tested patients, followed by direct parasitological examination and histopathology. Serological methods (indirect immunofluorescence reaction; enzyme-linked immunosorbent assay) are not yet officially used in Brazil to diagnose CL^2^. The definitive diagnosis is obtained by demonstrating the parasites in direct parasitological examination with material obtained from the lesions, or by visualizing them in histological sections or by cultures of aspirates or fragments of a skin or mucosal lesion. Direct parasitological examination is low-cost and easy to perform^32^, but it essentially depends on the training of the technician who collects the material and analyzes it on the microscope. Our data showed that it is used in less than half of patients with CL in Rio de Janeiro State. The sensitivity of visualizing amastigote forms in histological examinations is considered low^33^. Immunohistochemistry demonstrates greater sensitivity than traditional hematoxylin-eosin staining for detecting the parasite^32^, but it is restricted to few reference centers in the country. Histological examination was performed in less than half of the patients reported in this study. Culture and molecular tests are not yet evaluated in the SINAN notification form. The isolation of the parasite from a lesion fragment sown in appropriate culture media enables the visualization of promastigote parasitic forms with good sensitivity and specificity^33,34^, but it is expensive, its performance varies depending on the laboratory in which it is carried out, and it is also restricted to a single reference center in Rio de Janeiro State. Diagnosis can also be obtained by molecular methods such as polymerase chain reaction (PCR)^33,35^, being particularly recommended when conventional methods are negative, but it is restricted to a single reference center in Rio de Janeiro city (Oswaldo Cruz Foundation).

In Brazil, CL must be notified via a specific disease form in SINAN, which is essential for medicines to be provided free of charge by the Brazilian Ministry of Health^2^. The notification enables access to medicines such as meglumine antimoniate, which is not commercially available. Other medications, such as liposomal amphotericin B, are costly and require a hospital environment for intravenous administration. This makes treatment performed outside the public health system too expensive.

The use of data from SINAN records provides reliable rates of CL occurrence. However, we have to consider the possibility of underreporting in areas with no primary health unity, or with precarious access to it. A limitation of the study is the incomplete data of some variables in the SINAN forms as reported by health professionals^36^, which particularly hinders the analysis of items such as clinical outcome and HIV coinfection. It should be added that the surveillance of CL cases occurs passively when the patient seeks the health unit or is referred to the public health unit after seeking private care.

## CONCLUSION

The CLCI showed there are variations in the risk of CL, with cities showing a higher risk that subsequently decreases, and then increases again later. However, the disease does not tend to disappear definitively, probably because, despite the strong urbanization in Rio de Janeiro State, the conditions for the access of infected sandflies to human hosts in the peripheral neighborhoods of the cities persist, or due to work conditions. CL in Rio de Janeiro State affects people predominantly residing in urban areas and mainly in the middle age groups, between 20 and 59 years. It also predominates in the male sex. ML was significantly associated with men, with 80% chance of them being more affected than women. Among the diagnostic tests, LST showed higher positivity (93.2%) than others. However, after its discontinuation since 2016, the diagnosis requires greater attention, since direct parasitological examination and histopathology are performed in less than half of the patients and have limited diagnostic performances. Direct parasitological examination was moderately positive (77.1%). This study can help to better understand the scenario of American Tegumentary Leishmaniasis, being a subsidy to guide the design of future control and prevention actions in Rio de Janeiro State.
